# Evaluation of a Modified Cefsulodin-Irgasan-Novobiocin Agar for Isolation of *Yersinia* spp

**DOI:** 10.1371/journal.pone.0106329

**Published:** 2014-08-29

**Authors:** Lai Kuan Tan, Peck Toung Ooi, Elisabeth Carniel, Kwai Lin Thong

**Affiliations:** 1 Microbiology Division, Institute of Biological Sciences, Faculty of Science, University of Malaya, Kuala Lumpur, Malaysia; 2 Department of Clinical Veterinary Studies, Faculty of Veterinary Medicine, University Putra Malaysia, Serdang, Malaysia; 3 *Yersinia* Research Unit, National Reference Laboratory and WHO Collaborating Centre for *Yersinia*, Institut Pasteur, Paris, France; University of Helsinki, Finland

## Abstract

*Y. enterocolitica* and *Y. pseudotuberculosis* are important food borne pathogens. However, the presence of competitive microbiota makes the isolation of *Y. enterocolitica* and *Y. pseudotuberculosis* from naturally contaminated foods difficult. We attempted to evaluate the performance of a modified Cefsulodin-Irgasan-Novobiocin (CIN) agar in the differentiation of *Y. enterocolitica* from non-*Yersinia* species, particularly the natural intestinal microbiota. The modified CIN enabled the growth of *Y. enterocolitica* colonies with the same efficiency as CIN and Luria-Bertani agar. The detection limits of the modified CIN for *Y. enterocolitica* in culture medium (10 cfu/ml) and in artificially contaminated pork (10^4^ cfu/ml) were also comparable to those of CIN. However, the modified CIN provided a better discrimination of *Yersinia* colonies from other bacteria exhibiting *Yersinia*-like colonies on CIN (H_2_S-producing *Citrobacter freundii*, *C. braakii*, *Enterobacter cloacae*, *Aeromonas hydrophila*, *Providencia rettgeri*, and *Morganella morganii*). The modified CIN exhibited a higher recovery rate of *Y. enterocolitica* from artificially prepared bacterial cultures and naturally contaminated samples compared with CIN. Our results thus demonstrated that the use of modified CIN may be a valuable means to increase the recovery rate of food borne *Yersinia* from natural samples, which are usually contaminated by multiple types of bacteria.

## Introduction

*Yersinia* are Gram-negative bacteria belonging to the family *Enterobactericeae*. Among many species of *Yersinia*, *Y. enterocolitica* and *Y. pseudotuberculosis* are food borne pathogens and are a frequent cause of human yersiniosis [1]. *Y. enterocolitica* is one of the ten most important food borne enteric pathogens in Europe and the United States [1], [2]. *Y. enterocolitica* infections usually results from the consumption of contaminated food or water, and the disease tends to affect young children [1], [3]. The symptoms of yersiniosis include acute gastroenteritis with fever, bloody diarrhoea, and pseudo-appendicitis, which frequently leads to unnecessary laparotomy [4]. *Y. pseudotuberculosis* infections are less frequent than *Y. enterocolitica* infections. They are more common in adults and typically cause lower right abdominal pain that resembles appendicitis and, less frequently, diarrhea [1].

The isolation of *Yersinia* is laborious and difficult because the bacteria are easily outgrown by other microorganisms [5]. Selective enrichment [6]–[8], post-enrichment alkaline treatment [7], [9], [10], and selective agar isolation [6], [8] are the conventional methods for *Yersinia* isolation. The confirmation of the cultures is often accompanied by a series of biochemical tests, biotyping, serotyping, Polymerase Chain Reaction (PCR), and DNA sequencing of housekeeping genes [6], [8], [11]. However, the lack of a reliable and effective isolation method masks and underestimates the actual incidence of yersiniosis. The recovery of food borne *Yersinia* depends on several factors, such as (i) the amount of background microbiota present in the sample, (ii) the amount of microbiota that survives through selective enrichment and plating [5], and (iii) the amount of *Yersinia* present in the sample [12].

Several selective media have been developed for *Yersinia* isolation. The most frequently used medium is Cefsulodin-Irgasan-Novobiocin (CIN) [13]. CIN has been shown to be more specific than other conventional selective agars, such as *Salmonella-Shigella* (SS), MacConkey (MAC), Cellobiose-Arginine-Lysine (CAL), pectin agars, and other lactose-containing media, for the differentiation of *Yersinia* spp. from contaminating bacteria [14]. However, one of the weaknesses of CIN is that it fails to distinguish *Yersinia* spp. from several other mannitol-fermenting bacterial species, such as *Serratia liquefaciens*, *Enterobacter agglomerans*, *Aeromonas* spp., *Citrobacter* spp., *Providencia* spp. *Acinetobacter johnsonii, Achromobacter xylosoxidans* because all appear as a red “bull's eye” on CIN plates [14], [15]. Additional biochemical tests, such as esculin, phenylalanine deaminase, arginine dihydrolase, hydrogen sulphide, urease, and lysine decarboxylase, are needed to further differentiate *Yersinia* spp. from other *Yersinia*-like bacterial species [16]. These post-isolation steps are laborious, time consuming, and costly.

Some researchers have modified the existing selective agars to improve the isolation of virulent *Y. enterocolitica*. Fukushima modified CIN agar by the addition of 0.1% esculin and 0.05% ferric citrate [17]. This modified medium, which is named Virulent *Yersinia enterocolitica* (VYE) agar, allows the differentiation of pathogenic *Y. enterocolitica* colonies that do not hydrolyse esculin from non-pathogenic esculin-positive colonies. However, esculin hydrolysis produces a dark-brown diffusible pigment on VYE agar that can mask esculin-negative colonies, including virulent *Y. enterocolitica* [18], [19]. Furthermore, other mannitol-fermenting non-esculin-hydrolysing species cannot be distinguished from virulent *Y. enterocolitica*. Differentiation based on the size of the colonies of different bacteria on both CIN and VYE agars is also ineffective because *Y. enterocolitica* tends to grow more slowly and its colonies are smaller when in competition for space and nutrients.

Some researchers prefer to use the Statens Serum Institut (SSI) enteric medium for the detection of *Yersinia* spp. from highly contaminated samples, such as faeces, because it allows the identification of a range of enteric pathogens [20], [21]. However, SSI is reported to have a lower selective effect compared with CIN agar and requires at least 3×10^6^ *Yersinia* cfu/g of faeces for successful recovery [21]. This indicates that the SSI medium may provide false-negative results if the naturally occurring *Yersinia* is below the detection limit.

Chromogenic-based media have become increasingly popular in recent years for the isolation of enterobacteria. To date, three chromogenic media have been developed for the specific detection of virulent *Y. enterocolitica*: the *Y. enterocolitica* chromogenic medium (YeCM), [19] *Y. enterocolitica* agar (YECA) [18], and CHROMagar *Yersinia* (CAY) [15]. All three media allow the differentiation of virulent *Y. enterocolitica* from non-virulent strains and from other enterobacteria. However, YeCM and YECA are non-marketed media and require laborious and laboratory-made preparation. Moreover, all three media are relatively expensive, which seriously limits their routine usage. In addition, false-positive results from the chromogenic medium have been reported due to wrong colour interpretation by the user [22]. The purple, light denim blue, and light pink colours are frequently miss-interpreted as mauve and cannot be ruled out as possible presumptive colonies [22]. Furthermore, even if the chromogenic media are useful for differentiating the virulent from the non-virulent biotypes, the use of CIN is still required for the isolation step prior to plating on chromogenic media [23].

Thus, a medium that is less expensive than chromogenic media and improves the differentiation of *Yersinia* spp. from contaminating bacteria would thus be of value for routine use in microbiology laboratories. Therefore, the main objective of this study was to evaluate the performance of modified CIN agar for the differentiation of *Yersinia* spp. from other natural microbiota, such as H_2_S-producing *C. freundii*, *C. braakii*, *E. cloacae*, *A. hydrophila*, and *P. rettgeri*. The plating efficiencies, detection limits, and recovery strengths of CIN and modified CIN in pure culture, artificial contaminated raw pork meat, artificially prepared bacterial mixtures, and natural contaminated samples were evaluated and compared.

## Materials and Methods

### Modification of CIN agar

The modified CIN agar was prepared by the addition of 1% L-arginine (Sigma, Germany), 0.8 g/L ferric ammonium citrate (BDH Prolabo, UK), 6.8 g/L sodium thiosulphate (BDH Prolabo), and 2.0 g/L DL-phenylalanine (Sigma) at pH 7.4±0.02 to the CIN Agar Base (Oxoid, UK).

### Plating efficiency of CIN and modified CIN

The plating efficiencies of CIN and modified CIN agar were evaluated using the 50 bacterial strains listed in Table 1. These include *Yersinia* spp., other *Enterobacteriaceae*, selected Gram-negative and Gram-positive bacteria.

**Table 1:** Colony morphology of *Yersinia* spp. and other bacteria on CIN and modified CIN.

Bacterial species (strain number)	Colony morphology on:		
	CIN^a^ (ae^b^)	Modified CIN (ae)	Modified CIN (mic^c^)
***Yersinia enterocolitica***			
bioserotype 1A/O:6,30 (IP102)	NG^d^	NG	NG
bioserotype1A/O:5 (PC-M16-2)	Rbe	Rbe	Rbe
bioserotype 1B/O:8 (IP11105, ATCC 9610, YE036c-CY)	Rbe^e^	Rbe	Rbe
bioserotype 2/O:9 (IP383)	Rbe	Rbe	Rbe
bioserotype 3/O:1,2,3 (IP135)	Rbe	Rbe	Rbe
bioserotype 3 variant/O:3 (PC-M1-K1)	Rbe	Rbe	Rbe
bioserotype 4/O:3 (IP134)	Rbe	Rbe	Rbe
bioserotype 5/O:2,3 (IP178)	Rbe	Rbe	Rbe
**Other ** ***Yersinia* spp.**			
*Y. aldovae* (IP6005)	Rbe	Rbe	Rbe
*Y. bercovieri* (IP3443)	Rbe	Rbe	Rbe
*Y. frederiksenii* (IP3842)	Rbe	Rbe	Rbe
*Y. intermedia* (IP955)	Rbe	Rbe	Rbe
*Y. kristensenii* (IP105)	Rbe	Rbe	Rbe
*Y. mollaretii* (IP33766)	Rbe	Rbe	Rbe
*Y. pseudotuberculosis* (IP34476)	Rbe	Rbe	Rbe
**Other ** ***Enterobacteriaceae***			
*Citrobacter,*			
* freundii*, H_2_S-producing (YC-K1-3)	Rbe	Rbe + Bc^f^	Rbe + Bc
* freundii*, non-H_2_S-producing (YC-S1-5, YC-T1-1)	Rbe	Rbe	Rbe
* braakii* (YC-T1-K1)	Rbe	Rbe + Bc	Rbe + Bc
* koseri* (YC-VG2-1)	Rbe	Rbe	Rbe
*Providencia rettgeri* (IC-PP2a-9, IC-PP3a-K1, IC-PP6a-10)	Rbe	Rbe + Bp	Rbe + Bp
*Enterobacter cloacae* (YC-I1-K1, YC-I1-K2, YC-I1-K3)	Rbe	P^g^	P
*Pantoae* spp. (PI-TSP30a-K1)	Rbe	Rbe	Rbe
*Serratia,*			
* odorifera* (PC-TSP36b-3)	Rbe	Rbe	Rbe
* marcescens* (YC-M2-11)	Rbe	Rbe	Rbe
*Morganella morganii* (YS-TSP7b-1)	C^h^	C + Bp	C + Bp
*Salmonella*,			
Paratyphi A (ATCC 9150)	NG	NG	NG
Paratyphi B (ATCC 8759)	NG	NG	NG
Paratyphi C (ATCC 9068)	NG	NG	NG
Typhimurium (ATCC 13311)	NG	NG	NG
Typhi (ATCC 6539)	NG	NG	NG
* enterica* (ATCC 10376)	NG	NG	NG
*Escherichia coli* (ATCC25922, O157:H7)	NG	NG	NG
*Shigella sonnei* (ATCC 11060)	NG	NG	NG
*Proteus penneri* (IS-TSP7b-3)	NG	NG	NG
**Other Gram-Negative Bacteria**			
*Aeromonas hydrophila* (Ae 20)	Rbe	P + Bp^i^	P + Bp
*Vibrio* spp. (VSP-C12-1210, VS-A29-0810)	NG	NG	NG
*Pseudomonas aeruginosa* (ATCC 9027)	NG	NG	NG
**Gram-Positive Bacteria**			
*Enterococcus faecalis* (ATCC 29212)	NG	NG	NG
*Listeria monocytogenes* (ATCC 7644)	NG	NG	NG
*Staphylococcus aureus* (ATCC 6538, MRSA 0807-1)	NG	NG	NG

^*a*^CIN, Cefsulodin-Irgasan-Novobiocin.

^*b*^ae, aerobic.

^*c*^mic, microaerophilic.

^*d*^NG, no growth.

^*e*^Rbe, red bull's eye.

^*f*^Bc, black centre.

^*g*^P, pink.

^*h*^C, colourless.

^*i*^Bp, brown diffusible pigment.

IP, Institut Pasteur, strain collection of the French *Yersinia* Reference laboratory.

ATCC, American Type Culture Collection.

Others, strain collection of the Laboratory of Biomedical Science and Molecular Microbiology, Institute of Graduate Studies, University of Malaya, Malaysia.

The bacterial strains retrieved from the −20°C glycerol stocks were grown on Brain Heart Infusion agar (BD, USA). *Yersinia* strains were incubated at 25°C for 24–48 h, whereas other bacteria were incubated overnight at 37°C. Using needle inoculators, fresh bacterial cultures were dotted or streaked on CIN or modified CIN agar and incubated under aerobic or microaerophilic conditions at 25°C for 24–48 h. Microaerophilic conditions were created by placing the inoculated agar plates in candle jars to facilitate the visualization of H_2_S-producing bacteria [24]. The plating efficiencies on CIN and modified CIN agar were determined by screening for the presence of colonies with the expected *Yersinia* morphology [red centre with colourless translucent rim (red bull's eye)].

### Limit of detections (LODs) of CIN and modified CIN for *Y. enterocolitica* strains

The LOD was determined using the method recommended by the Microbiological Methods Committee [25]. *Y. enterocolitica* was chosen as representing strain. Overnight *Y. enterocolitica* cultures were serially diluted from 10^8^ to 10^1^ colony forming units (cfu)/ml and spread onto CIN and modified CIN agar. The plating efficiencies were determined six times using independent cell suspension. The plates were incubated under aerobic conditions at 25°C for 24–48 h. In addition, one set of samples on modified CIN agar was incubated under microaerophilic conditions at 25°C for 24–48 h. The LOD was defined as the lowest concentration of *Y. enterocolitica* with culturable bacteria detectable in at least 50% of the replicates (as low as one colony detectable in each replicate, and at least three out of six positive replicates).

### Quantification of *Y. enterocolitica* growth on CIN and modified CIN compared with that on LBA

The growth of *Y. enterocolitica* on CIN and modified CIN was compared to that on Luria-Bertani Agar (LBA)(Oxoid, USA). *Y. enterocolitica* suspension was adjusted to approximately 10^3^ cfu/ml and plated on LBA, CIN, and modified CIN. The mean cfu (six replicates) of *Y. enterocolitica* on LBA, CIN, and modified CIN was calculated after incubation at 25°C for 48 h. The results were expressed as the percentages of mean cfu on CIN/LBA and on modified CIN/LBA for each incubation condition [21].

### LOD and recovery rate of *Y. enterocolitica* in artificially contaminated raw pork meat on CIN and modified CIN

The effect of the natural microbiota on the recovery of *Y. enterocolitica* from a food matrix (with or without stress treatment, kept at −20°C for three weeks after bacterial spiking) was studied [25]. An artificially contaminated raw pork meat was used because yersiniosis is frequently associated with the consumption of contaminated pork products. For food matrix without stress treatment, approximately 1.5 kg of freshly purchased minced pork meat was processed immediately following spiking. Cell suspensions of *Y. enterocolitica* were serially diluted as previously described and 250 µL of each suspension was mixed with 25 g of minced meat homogenised in 24.75 mL of phosphate buffered saline (PBS, Sigma) to obtain final concentrations ranging from 10^1^ to 10^8^ cfu/ml. The meat suspensions were homogenised manually for 30 s and incubated at 25°C for 30 min. An aliquot of each homogenate was then plated on CIN and modified CIN agar, and the cultures were incubated under aerobic conditions at 25°C for 24–48 h. The same procedures were repeated for a set of samples on modified CIN agar incubated under microaerophilic conditions at 25°C for 24–48 h. Six replicates were performed for each bacterial concentration. The mean cfu/ml of the background microbiota in the un-inoculated pork meat (25 g of pork homogenised in 25 mL of PBS, six replicates) was determined after plating on LBA.

The LOD of *Y. enterocolitica* in the artificially contaminated pork meat was defined as the lowest concentration of culturable *Y. enterocolitica* detectable in at least 50% of the replicates (as low as one colony detectable on each replicate, and at least three out of six positive replicates) [25]. The ratio of the mean cfu of *Y. enterocolitica* to the background microbiota was also determined.

The same spiked minced meat was subjected to stress treatment by keeping the spiked food samples at −20°C for two weeks after bacterial spiking (to mimic food storage conditions) [25]. The same plating procedures were then performed. The percentage of *Y. enterocolitica* colonies recovered from the food (with and without stress treatment) was determined by the selection of representative colonies from each plate that were identified through the PCR targeting of the *Y. enterocolitica*-specific 16S rRNA and *ail* genes, as described previously [11]. The primer pairs used were Y1 (5′-AATACCGCATAACGTCTTCG-3′) and Y2 (5′-CTTCTTCTGCGAGTAACGTC-3′) for the *Y. enterocolitica*-specific 16S rRNA gene and A1 (5′ –TTAATGTGTACGCTGCGAGTG-3′ and A2 (5′-GGAGTATTCATATGAAGCGTC-3′) for the *ail* gene. The PCR amplicons were purified using the MEGA-spin Agarose Gel Extraction Kit (*iNtRON* Biotechnology, Korea) and confirmed by DNA sequencing.

### Determination of the recovery of *Y. enterocolitica* from artificial bacterial mixtures

Overnight cultures of *Y. enterocolitica* IP135 and bacteria exhibiting *Yersinia*-like colonies on CIN agar (H_2_S-producing *C. freundii*, *C. braakii*, *A. hydrophila*, *P. rettgeri*, and *E. cloacae*) were adjusted to a concentration of 10^4^ cfu/ml and mixed together. Six independent pooled bacterial suspensions were prepared, and spread on CIN and modified CIN agar, and incubated aerobically at 25°C for 24–48 h. Presumptive *Y. enterocolitica* colonies were isolated from each replicate and subjected to PCR confirmation [11] to determine the percentage of *Y. enterocolitica* on both media.

### Determination of the recovery rate of *Y. enterocolitica* in naturally contaminated samples

A total of 52 rectal swabs from swine were tested for the presence of *Y. enterocolitica* using different enrichment procedures and plating on CIN and modified CIN agars (Table 2, methods 1 to 6). Briefly, all of the swab specimens were (i) directly streaked onto the selective medium (methods 1 and 2), (ii) subjected to cold enrichment (incubation at 4°C for three weeks in PBS) before being plated on CIN and modified CIN (methods 3 and 4) [8], and (iii) subjected to cold enrichment and then alkaline treatment before being plated onto the two selective media (methods 5 and 6) [7], [8], [9], [10]. Presumptive *Y. enterocolitica* colonies were isolated and subjected to PCR confirmation [11]. In parallel to the isolation procedures, PCR detection of *Y. enterocolitica* was carried out directly on the boiled cell lysates (99°C for 5 min) of the 52 rectal swabs samples following post-cold enrichment. The isolated *Y. enterocolitica* isolates was biotyped by using biochemical tests as described by Wauters et al. [26] and serotyped by using the O-Antisera “SEIKEN” set purchased (DENKA SEIKEN Co., Ltd, Japan).

**Table 2:** Recovery rate of *Y. enterocolitica* from the 52 naturally contaminated rectal swabs from swine.

Methods	No. of positive specimens recovered by plating (%)
Method 1 — Direct streaking onto CIN^a^	0 (0)
Method 2 — Direct streaking onto mCIN^b^	0 (0)
Method 3 — PBS^c^-CIN	0 (0)
Method 4 — PBS-mCIN	2 (3.8)
Method 5 — PBS-KOH^d^-CIN	0 (0)
Method 6 — PBS-KOH-mCIN	2 (3.8)

The prevalence of *Y. enterocolitica* determined by using a duplex PCR targeting the *Y. enterocolitica*-specific 16S rRNA and *ail* genes [11] was 2/52 (3.8%).

^*a*^CIN, Cefsulodin-Irgasan-Novobiocin.

^*b*^mCIN, modified CIN.

^*c*^PBS, phosphate buffered saline, a cold enrichment at 4°C for three weeks [8].

^*d*^KOH, post-enrichment alkaline treatment.

### Statistical analysis

The statistical significance was calculated by the Student's t-test, Chi-square test, or Fisher's test (when appropriate) using the R software (version 2.12.2). The difference was considered significantly if *p*<0.05.

### Ethics Statement

All of the rectal swab samples from swine were collected with supervision from the institution veterinarian during routine farm visits. No animal was sacrificed or injured during the sampling procedure. The sampling complied with the current guidelines for the care and use of animals and was approved by the Animal Care and Use Committee (ACUC, AUP number: T006/2013), Faculty of Veterinary Medicine, Universiti Putra Malaysia.

## Results

### Growth characteristics and colony morphology on CIN and modified CIN agar

**Figure 1:**
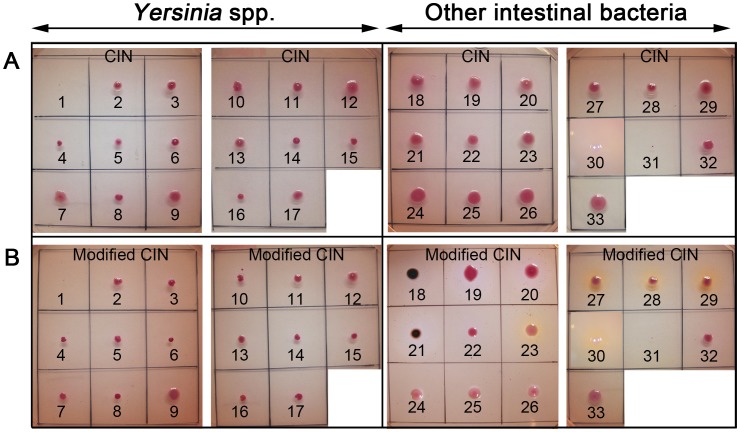
Bacteria on CIN (A) *and modified CIN (B)*. 1, *Y. enterocolitica* 1A/O:6,30 (IP102); 2, *Y. enterocolitica* 1B/O:8 (IP11105); 3, *Y. enterocolitica* 2/O:9 (IP383); 4, *Y. enterocolitica* 3/O:1,2,3 (IP135); 5, *Y. enterocolitica* 4/O:3 (IP134); 6, *Y. enterocolitica* 5/O:2,3 (IP178); 7, *Y. enterocolitica* 1B/O:8 (ATCC 9610); 8, *Y. enterocolitica* 3/O:3 (PC-M1-K1); 9, *Y. enterocolitica* 1A/O:5 (PC-M16-2); 10, *Y. aldovae* (IP6005); 11, *Y. bercovieri* (IP3443); 12, *Y. frederiksenii* (IP3842); 13, *Y. intermedia* (IP955); 14, *Y. kristensenii* (IP105); 15, *Y. mollaretii* (IP33766); 16, *Y. pseudotuberculosis* (IP34476); 17, *Y. enterocolitica* 1B/O:8 (YE036c-CY); 18, *C. freundii*, H_2_S-producing; 19, 20, *C. freundii*, nonxH_2_S-producing; 21, *C. braakii*; 22, *C. koseri*; 23, *A. hydrophila*; 24, 25, 26, *E. cloacae*; 27, 28, 29, *P. rettgeri*; 30, *M. morganii*; 31, *Pantoae* spp.; 32, *S. odorifera*; 33, *S. marcescens*.

The capability of bacteria to grow on CIN and modified CIN agar and the colony morphology of 50 bacterial strains were determined. Both CIN and modified CIN allowed the growth of all *Yersinia* strains tested with the exception of the non-pathogenic strain IP102 (*Y. enterocolitica* bioserotype 1A/O:6,30), which was inhibited on both media. Furthermore, all *Yersinia* colonies displayed the same characteristic red bull's eye feature (Table 1 and Figure 1, Nb 2–17). Therefore, the CIN modification did not alter the growth and colony shape of *Yersinia* strains. The *Y. enterocolitica* colonies on modified CIN were tiny (≤0.5 mm) and only exhibited the characteristic red bull's eye morphology after 30 h of incubation, whereas this typical shape was visible after 24 h on CIN. This observation indicates that the modified CIN plates should not be read before 30 h.

The CIN and modified CIN exhibited the same growth inhibitory effect on various species of *Salmonella, E. coli, Shigella, Proteus*, *Vibrio, Pseudomonas, Enterococcus, Listeria*, and *Staphylococcus* (Table 1). The two media allowed the growth of non-H_2_S-producing *C. freundii*, *C. koseri*, *S. odorifera*, *S. marcescens*, and *Pantoea* spp., which produced colonies with the red bull's eye morphology (Figure 1 and Table 1). Hence, the modification of CIN did not improve the differentiation of these bacteria from *Yersinia* spp.

In contrast, the modified CIN but not CIN allowed the differentiation of *Yersinia* spp. from several other *Enterobacteriaceae* and *A. hydrophila*. On the modified CIN, the colonies of *C. braakii* and H_2_S-producing *C. freundii* exhibited a black centre (Figure 1, Nb 18 and 21), the *P. rettgeri* colonies were surrounded by a brown diffusible pigment (Figure 1, Nb 27–29), the *E. cloacae* colonies were light pink (Figure 1, Nb 24–26), *A. hydrophila* appeared as pink colonies surrounded by a brown pigment (Figure 1, Nb 23), and *M. morganii* formed tiny colourless colonies surrounded by a brown pigment (Figure 1, Nb 30). The modified CIN is thus more efficient than CIN for the discrimination of *Yersinia* spp. from these *Yersinia*-like bacterial species.

Changing the incubation conditions for the modified CIN had no effect on the colony morphology of the various species tested (Table 1). However, the formation of a black centre in H_2_S-producing colonies could be observed under microaerophilic conditions even when their size was ≤1 mm, whereas the black centre appeared when the colonies were larger under aerobic conditions. Therefore, microaerophilic conditions improved the visualization of H_2_S-producing bacteria.

### LOD of CIN and modified CIN agar for *Y. enterocolitica* detection

The capacities of four *Y. enterocolitica* strains, which represented non-pathogenic (PC-M16-2), moderate pathogenic (IP383 and IP135), and highly pathogenic (ATCC 9610) strains, to grow on CIN and modified CIN agar were evaluated. Various cell suspensions (from 10^1^ to 10^8^ cfu/ml) were spread onto CIN and modified CIN agar, incubated for 24–48 h at 25°C, and then observed. In addition, one set of samples plated on modified CIN agar was incubated under microaerophilic conditions at 25°C for 24–48 h.

The LOD for all four *Y. enterocolitica* strains was 10 cfu/ml on both CIN and modified CIN under aerobic conditions (Table S1). Although the LOD for strain ATCC 9610 was slightly higher on the modified CIN under microaerophilic (10^2^ cfu/ml) compared with aerobic conditions (10^1^ cfu/ml), the difference in sensitivity for all four strains tested under these different conditions was not significant (Student t-test, *p*>0.05). Therefore, the use of microaerophilic conditions did not alter the growth of *Y. enterocolitica* on modified CIN.

### Quantification of *Y. enterocolitica* growth on CIN and modified CIN compared with that on LBA

The ability of CIN and modified CIN to support the growth of *Y. enterocolitica*, compared with that of LBA was evaluated. The growth of four *Y. enterocolitica* strains (IP383, IP135, ATCC 9610, and PC-M16-2) on LBA, CIN, and modified CIN was quantified and is presented as percentages of the mean cfu on CIN or modified CIN versus that on LBA. Values between 80% and 120% were considered to indicate growth efficiency on CIN or modified CIN similar to that on LBA (100%) [21].

As shown in Table 3, the numbers of *Y. enterocolitica* colonies recovered on CIN, modified CIN, and LBA were similar, regardless of whether the bacteria were grown under aerobic or microaerophilic conditions (the percentages of mean colony count on CIN or modified CIN versus LBA were between 80% and 120%). Therefore, the different media and conditions allowed the growth of *Y. enterocolitica* colonies with the same efficiency.

**Table 3:** Growth of *Y. enterocolitica* on CIN and modified CIN under different incubation conditions compared with that on LBA (control medium).

Strains	Bioserotype	Percentage of cfu (%)			
		Aerobic		Microaerophilic	Aerobic/Microaerophilic
		CIN^a^/LBA^b^	mCIN^c^/LBA	mCIN/LBA	mCIN
PC-M16-2	1A/O:5	110.3	108.9	106.8	102.0
ATCC 9610	1B/O:8	88.5	84.3	84.3	100.0
IP383	2/O:9	109.6	101	94.5	106.9
IP135	3/O:1,2,3	96.7	82.1	82.4	99.6

^*a*^CIN, Cefsulodin-Irgasan-Novobiocin.

^*b*^LBA, Luria-Bertani agar.

^*c*^mCIN, modified CIN.

### LOD of *Y. enterocolitica* from artificially contaminated raw pork meat

The ability of CIN and modified CIN to recover *Y. enterocolitica* from a food matrix was determined using raw pork meat (with or without stress treatment, kept at −20°C, three weeks after bacterial spiking [25]) mixed with various concentrations (from 10^1^ to 10^8^ cfu/ml) of *Y. enterocolitica* IP135. The IP138 strain was chosen because it is of biotype 3 and this particular biotype has been frequently reported in food and animal from Asia and Southeat Asia countries with warm climate (Southern China, Malaysia, Taiwan and Thailand) [30], [31], [32], [33]. The ratio of LOD to background microbiota was calculated by dividing the mean cfu of the LOD to the mean cfu of the un-inoculated pork meat.

The LOD of strain IP135 in both raw pork meat with and without stress treatment was 10^4^ cfu/ml on both media incubated under aerobic conditions (Table S2), indicating that the modification of CIN did not impair its capability to recover *Y. enterocolitica* from the studied food matrix. The finding that this LOD was 100-fold higher than that of the bacteria grown in pure culture (Table S1, 10 cfu/ml) confirms the impact of the presence of background microbiota on the recovery of *Y. enterocolitica*. At the LOD value of 10^4^ cfu/ml, the food homogenates contained a ratio of *Y. enterocolitica* to microbiota of 1∶412 (Table S2), indicating that *Y. enterocolitica* colonies cannot be successfully identified in the meat sample if it is below this ratio.

For the food matrix with stress treatment (kept at −20°C for three weeks after bacterial spiking) and incubated under microaerophilic conditions, the LOD decreased to 10^3^ cfu/ml on modified CIN (Table S2), which suggests that this incubation condition may slightly increase the recovery rate of *Y. enterocolitica* in food since visualization of H_2_S-producing bacteria was improved.

### Differentiation of *Y. enterocolitica* colonies from enterobacteria exhibiting *Yersinia*-like morphology on CIN

Suspensions containing similar concentrations (10^4^ cfu/ml) of *Y. enterocolitica* (IP135) and other enterobacterial species exhibiting *Yersinia*-like colonies on CIN agar, such as H_2_S-producing *C. freundii*, *C. braakii*, *E. cloacae*, *P. rettgeri*, and *A. hydrophila*, were plated on CIN and modified CIN to compare the number of true *Y. enterocolitica* recovered on each medium. Presumptive colonies of *Y. enterocolitica* (red bull's eye morphology) were picked and analysed by PCR for confirmation.

Only the modified CIN but not CIN allowed the differentiation of *Y. enterocolitica* (IP135) from H_2_S-producing *C. freundii*, *C. braakii*, *E. cloacae*, *P. rettgeri*, and *A. hydrophila*. The percentages of true *Y. enterocolitica* (IP135) recovered from the bacterial mixtures were 33.3% on CIN and 60.0% on modified CIN (Table 4), and the difference was significant (Chi-square test, *p*<0.05). Therefore, the use of modified CIN agar enhanced the differentiation of *Yersinia* colonies from those of *Yersinia*-like species.

**Table 4:** Recovery of *Y. enterocolitica* from artificially prepared bacterial mixtures and artificially contaminated raw pork.

Experiments	Agar	Number of positive *Y. enterocolitica*		
		True^a^ (%)	False^a^ (%)	Total
IP135 + bacterial mixture^b^	CIN^c^	20 (33.3)	40 (66.7)	60
	Modified CIN	36 (60.0)	24 (40.0)	60
IP135 + background microbiota from raw pork	CIN	43 (62.3)	26 (37.7)	69
	Modified CIN	50 (72.5)	19 (27.5)	69

a True and false positive *Y. enterocolitica* colonies were determined by using a duplex PCR targeting the *Y. enterocolitica*-specific 16S rRNA and *ail* genes [11].

b Mixture of bacteria exhibiting *Yersinia*-like colonies on CIN agar (*C. freundii, C. braakii, E. cloacae, P. rettgeri, A. hydrophila*).

c CIN, Cefsulodin-Irgasan-Novobiocin.

In artificially contaminated raw pork, the percentage of true *Y. enterocolitica* (IP135) colonies also increased from 62.3% on CIN to 72.5% on modified CIN (Table 4), however this difference was not statistically significant (Chi-square test, *p*>0.05).

### Determination of the recovery of *Y. enterocolitica* from naturally contaminated samples

The efficiency of CIN and modified CIN agars for the recovery of *Y. enterocolitica* from the 52 naturally contaminated samples (rectal swabs from swine) was evaluated and compared in three ways: (i) after direct plating on the agars (methods 1 and 2); (ii) after cold enrichment followed by plating on the agars (methods 3 and 4); (iii) after cold enrichment, alkaline treatment, and plating on the agars (methods 5 and 6) (Table 2). The detection of *Y. enterocolitica* by PCR in post-PBS enrichment broths was 2/52 (3.8%). Both modified CIN and CIN did not recover any *Y. enterocolitica* from the samples using the direct plating method (methods 1 and 2). After cold enrichment (methods 3 and 4), the modified CIN allowed the recovery of *Y. enterocolitica* from all PCR-positive samples (2/52, 3.8%), while no *Y. enterocolitica* was identified on CIN (Table 2). Cold enrichment and alkaline treatment followed by plating on modified CIN (methods 5 and 6) also allowed the isolation of *Y. enterocolitica* from all PCR-positive samples (2/52, 3.8%), while no *Y. enterocolitica* was detected on CIN (Table 2). The results showed that modified CIN resulted in the detection of a larger number of positive samples than CIN for the recovery of *Y. enterocolitica* from naturally contaminated samples. The results of biotyping and serotyping indicated that all the *Y. enterocolitica* isolated were of bioserotype 3/O:3 variant (VP negative).

## Discussion

*Y. enterocolitica* and *Y. pseudotuberculosis* are important food borne pathogens that cause human yersiniosis worldwide, and the incidence of yersiniosis is vastly underestimated. This underestimation may occur because the infection is self-limiting in most cases. The lack of a good isolation medium makes the recovery of *Yersinia* difficult. In the search for a medium that would allow a better discrimination of *Yersinia* colonies from other bacterial spp. while maintaining the selective properties of CIN, various chemical components have been added to this medium to detect three biochemical activities: phenylalanine deaminase, arginine dihydrolase, and H_2_S production. According to Bergey et al. [27], *C. braakii*, H_2_S-producing *C. freundii*, *E. cloacae*, *P. rettgeri*, and *A. hydrophila* can be differentiated from *Yersinia* based on their biochemical utilisation of these components.

Ferric ammonium citrate and sodium thiosulphate are substrates for H_2_S production, which results in the formation of a black centre on bacterial colonies. Phenylalanine deaminase converts DL-phenylalanine in phenylpyruvate, which in the presence of iron (III) ions and citrate, forms a brown, diffusible pigment in agar around the bacterial colonies. The dihydrolysis of L-arginine produces an alkaline substrate that gives a yellow colour to bacterial colonies. The addition of these substrates did not alter the formation of the red bull's eye feature of *Yersinia* colonies, although the formation of *Y. enterocolitica* characteristic morphology was slightly delayed on modified CIN compared with CIN. Nevertheless, the plates could be read within 48 h (optimal incubation time for *Y. enterocolitica*) in the normal isolation step for *Yersinia* spp.

The differentiation of *Yersinia* from other mannitol-fermenting bacteria that exhibit *Yersinia*-like colonies CIN (H_2_S-producing *C. freundii*, *C. braakii, E. cloacae*, *A. hydrophila*, and *P. rettgeri*) was markedly easier on modified CIN compared with CIN. These bacterial species are naturally present in feces, raw food, and other environmental samples [27]. Because H_2_S-producing *C. freundii* and *C. braakii* ferment mannitol and produce H_2_S, these appeared as red bull's eye colonies with a black centre on modified CIN. The capacity of *E. cloacae* and *A. hydrophila* to ferment mannitol and dihydrolyse arginine resulted in the formation of pink colonies. Furthermore, *A. hydrophila* produces phenylalanine deaminase, which generates a diffusible brown pigment around the pink colonies. Similarly, *P. rettgeri* produces a phenylalanine deaminase and therefore appeared as red bull's eye colonies with a diffusible brown pigment. The diffusible brown pigment produced will not mask the appearance of non-phenylalanine deaminase-producing bacteria because this brown pigment has a light colour that is less intense than the dark-brown pigment produced in the VYE agar [17]. Van Damme et al. [28] reported that *Y. enterocolitica* forms small white colonies and not typical red bull's eyes colonies on CIN agar in the presence of an abundant background flora [28]. Similar results were observed in this study when the colony size of *Y. enterocolitica* was ≤0.5 mm on CIN. The tiny white/colourless *Y. enterocolitica* colonies obtained in this case resembled *M. morganii* on CIN, causing false-negative results during colony selection. A bonus benefit obtained with the modification of CIN was that *M. morganii* appeared as colourless colonies with a diffusible brown pigment on modified CIN due to their capacity to produce phenylalanine deaminase. Based on these differential metabolic properties, it was possible to eliminate a large number of bacterial colonies prior further biochemical testing. The addition of these chemicals in the modified CIN thus reduced the workload and additional costs associated with biochemical testing by decreasing the number of colonies to be tested and enhanced the detection rate by lowering the risk of selecting non-*Yersinia* colonies.

However, the modified CIN still has the limitation of not differentiating pathogenic *Y. enterocolitica* from non-pathogenic *Yersinia* species and from non-H_2_S-producing *C. freundii, C. koseri*, *Pantoea* spp., *S. odorifera*, and *S. marcescens*. Nevertheless, the combination usage of modified CIN with a chromogenic media, such as CHROMagar *Yersinia* [15] and YECA [18], and YeCM [19], may help eliminate non-pathogenic *Yersinia* without the need to conduct additional biochemical tests [23]. This combination usage may also reduce the false-positive results caused by wrong colour interpretations by the user [22].

**Figure 2:**
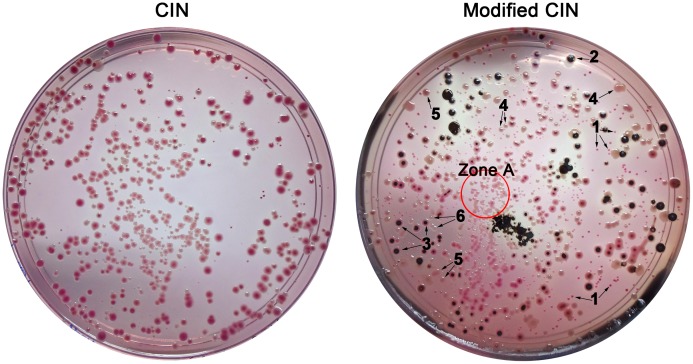
Colony morphology on CIN and modified CIN of artificially prepared bacterial mixtures. 1, *Y. enterocolitica* 3/O:1,2,3 (IP135); 2, *C. braakii*; 3, H_2_S-producing *C. freundii*; 4, *A. hydrophila*; 5, *P. rettgeri*; 6, *E. cloacae*. Zone A shows a region of bacterial clumping in which the formation of a black centre due to H_2_S production and of a brown diffusible pigment produced by a phenylalanine deaminase reaction could not be detected or could hardly be observed.

A better formation of a black centre by H_2_S-producing *C. freundii* and *C. braakii* colonies was observed when these were incubated under microaerophilic conditions. The reduction of sulphide to H_2_S gas is an anaerobic respiration and normally occurs in the middle of bacterial colonies [29]. Tiny colonies may not provide good anaerobic conditions for the production of H_2_S gas, and this phenomenon was observed in our samples (Zone A, Figure 2). The formation of a black centre could not be detected when the colonies were small and clumped together. Therefore, a longer incubation time (30 h to 48 h instead of 24 h) was required when the plates were incubated under normal (aerobic) conditions because bacteria grew bigger and fulfilled the anaerobic respiration requirement. Moreover, we observed that the formation of the brown diffusible pigment due to the phenylalanine deaminase reaction was hardly observable when the bacteria grew in clumps (Zone A) because the brown pigment diffused around the colony. Bacterial clumping may thus be a limitation of modified CIN for the visualisation of these bacteria. However, the clumped bacteria can be re-streaked on modified CIN for further identification.

The addition of chemicals did not inhibit the growth of the *Yersinia* strains tested, indicating that modified CIN has the same capacity as CIN to allow the growth of this species. It has been previously reported that CIN inhibits the growth of *Y. bercovieri* (formerly known as *Y. enterocolitica* bioserotype 3B/O:3) and some strains of *Y. pseudotuberculosis* due to the presence of cefsulodin in the medium [13], [15], [21], [34]. Because the amount of cefsulodin was not changed in the modified CIN, we expect similar results on this medium.

In evaluating the limit of detection (LOD) and influence of background microbiota of both CIN and modified CIN media, *Y. enterocolitica* was chosen as the representating bacterium. The LOD of the modified CIN for the detection of *Y. enterocolitica* (in pure cultures or in artificially contaminated raw pork meat) was comparable to that obtained on CIN. For pure *Y. enterocolitica* cultures, the LOD was 10 cfu/ml (for all four strains tested; IP383, IP135, ATCC 9610 and PC-M16-2). For artificially contaminated raw pork meat, *Y. enterocolitica* IP135 was chosen instead of a more common biotype 4 (e.g. IP134). This is because the biotype 3 has been frequently reported in food and animals (pigs and etc.) originated/imported from Asia and Southeat Asia countries with warm climate (Southern China, Malaysia, Taiwan and Thailand) [30], [31], [32], [33]. Whereas strains of biotype 4 are more commonly associated with humans infections in Europe and United States which is rarely reported in Southeast Asia [1], [2], [3], [6]. Based on the results in Table 1, all pathogenic *Y. enterocolitica* strains had similar growth characteristics and colony morphology on CIN and modified CIN agar, and strains of different biotypes also had similar LODs (Table S1). Hence only one representative strain (IP135, biotype 3) was used in the spiking experiments. The biotyping and serotyping results indicated that all *Y. enterocolitica* isolates were of bioserotype 3/O:3 variant (VP negative). This supports our speculation that this particular biotype is more common and supports the choice of *Y. enterocolitica* IP135 for our medium evaluation. The LOD of IP135 for artificially contaminated pork meat increased to 10^4^ cfu/ml because the presence of natural microbiota interfered with the recovery of *Y. enterocolitica*. Bacteria such as *C. freundii, C. amalonaticus, C. diversus, Hafnia alvei, Klebsiella pneumoniae, E. agglomerans, P. rettgeri,* and environmental *Yersinia* species that are naturally present in food may indeed have an inhibitory effect on the growth of *Y. enterocolitica* [34]. In a recent study conducted by Savin et al. [21], CIN was reported to be more efficient (LOD  = 3×10^3^ cfu/g of faeces) as compared to SSI medium (LOD  = 3×10^6^ cfu/g faeces) in isolating *Y. enterocolitica*. Therefore, we anticipate the modified CIN would be a useful medium in differentiating *Y. enterocolitica* from contaminated samples such as faeces.

Our investigation on the influence of background microbiota was done by three tests: (i) pool of bacterial mixture, (ii) artificially contaminated pork and (iii) naturally contaminated rectal swabs from swine. We observed that the *Y. enterocolitica* colonies were easily distinguished on modified CIN even when surrounded by *Yersinia-*like bacteria and background microbiota, but on CIN, it was much more difficult. The percentage of false-positive *Y. enterocolitica* recovered on CIN (66.7%) from an artificially prepared bacterial mixture can decrease to 40.0% on modified CIN, and the corresponding percentage from artificially contaminated raw pork meat can decrease from 37.7% on CIN to 27.5% on modified CIN (Table 4). At the same time, the true positive *Y. enterocolitica* isolates increased by nearly 27% and 10% on modified CIN compared to CIN in artificially prepared bacterial mixture and artificially contaminated pork, respectively (Table 4). The capability of modified CIN in reducing the number of false positive *Y. enterocolitica* isolates, especially in eliminating H_2_S-producing *Citrobacter* spp., *M. morganii, P. rettgeri, A. hydrophila, E. cloacae* can shorten the processing time and reduce the workload and costs associated with biochemical assays that were reported in many studies [13], [14], [15], [16], [23], [32]. Higher recovery rates from the modified CIN compared with that of CIN was further demonstrated using naturally contaminated samples (rectal swabs from swine, Table 2), indicating the modified CIN improved the recovery of *Y. enterocolitica* even on actual samples. The reason for the failure of CIN in isolating *Y. enterocolitica* from rectal specimens may due to the presence of high amount of *Yersinia*-like bacteria on CIN that caused the visualisation of the *Yersinia* difficult during plate analysis. In this study, the naturally contaminated samples were only enriched in one enrichment medium, PBS and our results indicated that a cold enrichment could help in recovering a larger number of positive samples than direct streaking method. A post-enrichment alkaline treatment showed no difference for the recovery rates of *Y. enterocolitica* (Table 2). The modification made on CIN enhanced the differentiating power of CIN while retaining the sensitivity (see results of LOD test) in isolating *Yersinia*. Hence, the modified CIN could also increase the recovery rates compared to CIN when it is used in combination with any other enrichment or isolating media that are reported to be useful in previous studies. For examples the irgarsan-ticarcillin-cholate (ITC)-CIN method [23], followed by streaking on YeCM and enrichment in peptone-sorbitol-bile (PSB) broth for 2 days [28] that are reported to be useful in isolation of pathogenic *Y. enterocolitica*.

Therefore, the use of modified CIN may significantly reduce the percentage of false-positive *Yersinia* recovered from a contaminated sample. The higher discriminatory power of the modified CIN compared with that of CIN was further demonstrated in artificially prepared bacteria mixture and naturally contaminated samples. Our results thus demonstrated that the use of modified CIN may be a valuable means to increase the recovery rate of *Yersinia* spp. from natural samples, which are usually contaminated by multiple types of bacteria.

## Supporting Information

Table S1 Growth efficiency and limit of detection of CIN and modified CIN for pure cultures of *Y. enterocolitica*. ^a^YE, *Yersinia enterocolitica*; ^b^CIN, Cefsulodin-Irgasan-Novobiocin; ^c^ae, aerobic; ^d^mCIN, modified CIN; ^e^mic, microaerophilic; ^f^LOD, limit of detection. The underlined numbers correspond to the LOD scores for each *Y. enterocolitica* strain. The LOD is defined as the lowest cfu/ml of culturable *Y. enterocolitica* detectable in at least 50% of the replicates seeded with *Y. enterocolitica*.
(DOCX)

Table S2 Growth efficiency and limit of detection of CIN and modified CIN for raw pork meat spiked with *Y. enterocolitica* IP135. ^a^MM, microbiota in the meat sample; ^b^CIN, Cefsulodin-Irgasan-Novobiocin; ^c^ae, aerobic; ^d^mCIN, modified CIN; ^e^mic, microaerophilic; ^f^LOD, limit of detection. The underlined numbers correspond to the LOD scores for each medium. The LOD is defined as the lowest cfu/ml of culturable *Y. enterocolitica* detectable in at least 50% of the replicates.
(DOCX)
