# Supplementary material for: Evaluation of a Modified Cefsulodin-Irgasan-Novobiocin Agar for Isolation of Yersinia spp
Source: PLoS One. 2014 Aug 29;9(8):e106329. doi: 10.1371/journal.pone.0106329 (PMC4149559; doi:10.1371/journal.pone.0106329)
Supplement: Table S1 — Growth efficiency and limit of detection of CIN and modified CIN for pure cultures of Y. enterocolitica. aYE, Yersinia enterocolitica; bCIN, Cefsulodin-Irgasan-Novobiocin; cae, aerobic; dmCIN, modified CIN; emic, microaerophilic; fLOD, limit of detection. The underlined numbers correspond to the LOD scores for each Y. enterocolitica strain. The LOD is defined as the lowest cfu/ml of culturable Y. enterocolitica detectable in at least 50% of the replicates seeded with Y. enterocolitica. (DOCX) [file pone.0106329.s001.docx]

Table S1. Growth efficiency and limit of detection of CIN and modified CIN for pure cultures of *Y. enterocolitica*.

|  | **Percentage of positive plates** | | | | | | | | | | | | | | |
| --- | --- | --- | --- | --- | --- | --- | --- | --- | --- | --- | --- | --- | --- | --- | --- |
|  | **YE 2/O:9**  **(IP383)** | | |  | **YE 3/O:1,2,3**  **(IP135)** | | |  | **YE 1B/O:8**  **(ATCC 9610)** | | |  | **YE 1A/O:5**  **(PC-M16-2)** | | |
| **Dilution of YE^a^ seeded on plate (cfu/ml)** | **CIN^b^ (ae^c^)** | **mCIN^d^ (ae)** | **mCIN (mic^e^)** |  | **CIN (ae)** | **mCIN (ae)** | **mCIN (mic)** |  | **CIN (ae)** | **mCIN (ae)** | **mCIN (mic)** |  | **CIN (ae)** | **mCIN (ae)** | **mCIN (mic)** |
| 10^8^ | 100 | 100 | 100 |  | 100 | 100 | 100 |  | 100 | 100 | 100 |  | 100 | 100 | 100 |
| 10^7^ | 100 | 100 | 100 |  | 100 | 100 | 100 |  | 100 | 100 | 100 |  | 100 | 100 | 100 |
| 10^6^ | 100 | 100 | 100 |  | 100 | 100 | 100 |  | 100 | 100 | 100 |  | 100 | 100 | 100 |
| 10^5^ | 100 | 100 | 100 |  | 100 | 100 | 100 |  | 100 | 100 | 100 |  | 100 | 100 | 100 |
| 10^4^ | 100 | 100 | 100 |  | 100 | 100 | 100 |  | 100 | 83 | 83 |  | 100 | 100 | 100 |
| 10^3^ | 100 | 100 | 100 |  | 100 | 100 | 100 |  | 100 | 100 | 67 |  | 100 | 100 | 100 |
| 10^2^ | 83 | 83 | 100 |  | 100 | 100 | 100 |  | 100 | 100 | 50 |  | 100 | 100 | 100 |
| 10^1^ | 83 | 83 | 100 |  | 100 | 100 | 100 |  | 100 | 67 | 33 |  | 100 | 100 | 100 |
|  |  |  |  |  |  |  |  |  |  |  |  |  |  |  |  |
| LOD^f^ (cfu/ml) | 10^1^ | 10^1^ | 10^1^ |  | 10^1^ | 10^1^ | 10^1^ |  | 10^1^ | 10^1^ | 10^2^ |  | 10^1^ | 10^1^ | 10^1^ |

^a^ YE, *Yersinia enterocolitica*

^b^ CIN, Cefsulodin-Irgasan-Novobiocin

^c^ ae, aerobic

^d^ mCIN, modified CIN

^e^ mic, microaerophilic

^f^ LOD, limit of detection

The underlined numbers correspond to the scores of LOD for each *Y. enterocolitica* strain. The LOD was defined as the lowest cfu/ml of culturable *Y. enterocolitica* detectable in at least 50% of the replicates seeded with *Y. enterocolitica*.
