# Supplementary material for: Evaluation of a Modified Cefsulodin-Irgasan-Novobiocin Agar for Isolation of Yersinia spp
Source: PLoS One. 2014 Aug 29;9(8):e106329. doi: 10.1371/journal.pone.0106329 (PMC4149559; doi:10.1371/journal.pone.0106329)
Supplement: Table S2 — Growth efficiency and limit of detection of CIN and modified CIN for raw pork meat spiked with Y. enterocolitica IP135. aMM, microbiota in the meat sample; bCIN, Cefsulodin-Irgasan-Novobiocin; cae, aerobic; dmCIN, modified CIN; emic, microaerophilic; fLOD, limit of detection. The underlined numbers correspond to the LOD scores for each medium. The LOD is defined as the lowest cfu/ml of culturable Y. enterocolitica detectable in at least 50% of the replicates. (DOCX) [file pone.0106329.s002.docx]

Table S2. Growth efficiency and limit of detection of CIN and modified CIN for raw pork meat spiked with *Y. enterocolitica* IP135.

| **Dilution of IP135 spiked**  **in 25 g of pork meat (cfu/ml)** | **Initial ratio** | **Without stress treatment** | | | **With stress treatment at**  **-20°C for two weeks** | | |
| --- | --- | --- | --- | --- | --- | --- | --- |
|  | **IP135:MM^a^** | **CIN^b^ (ae^c^)** | **mCIN^d^ (ae)** | **mCIN (mic^e^)** | **CIN (ae)** | **mCIN (ae)** | **mCIN (mic)** |
| 10^8^ | 1:0.0412 | 100 | 100 | 100 | 100 | 100 | 100 |
| 10^7^ | 1:0.412 | 100 | 100 | 100 | 100 | 100 | 100 |
| 10^6^ | 1:4.12 | 100 | 100 | 100 | 100 | 100 | 100 |
| 10^5^ | 1:41.2 | 100 | 100 | 100 | 100 | 100 | 100 |
| 10^4^ | 1:412 | 100 | 67 | 67 | 83 | 67 | 83 |
| 10^3^ | 1:4120 | 33 | 17 | 33 | 17 | 17 | 50 |
| 10^2^ | 1:41200 | 0 | 17 | 0 | 0 | 0 | 0 |
| 10^1^ | 1:412000 | 0 | 0 | 0 | 0 | 0 | 0 |
|  |  | 10^4^, 1:412 | 10^4^, 1:412 | 10^4^, 1:412 | 10^4^, 1:412 | 10^4^, 1:412 | 10^3^, 1:4120 |

^a^ MM, microbiota in the meat sample

^b^ CIN, Cefsulodin-Irgasan-Novobiocin

^c^ ae, aerobic

^d^ mCIN, modified CIN

^e^ mic, microaerophilic

^f^ LOD, limit of detection

The underlined numbers correspond to the LOD scores for each medium. The LOD was defined as the lowest cfu/ml of culturable *Y. enterocolitica* detectable in at least 50% of the replicates.
